# p53 and Ki-67 combined with periodic acid-Schiff staining for the diagnosis of early stage esophageal squamous cell carcinoma lesions in biopsy specimens

**DOI:** 10.1007/s10388-024-01102-7

**Published:** 2024-12-23

**Authors:** Feifei Liu, Hongying Zhao, Xue Li

**Affiliations:** Department of Pathology, Beijing Chao-Yang Hospital, Capital Medical University, Beijing, 100020 China

**Keywords:** Esophageal squamous cell carcinoma, Esophageal mucosa biopsy, P53, Ki-67, PAS staining, Early diagnosis, Retrospective study

## Abstract

**Background:**

Esophageal cancer is highly prevalent in China, predominantly represented by squamous cell carcinoma. This retrospective study sought to evaluate the diagnostic efficacy of four staining protocols in identifying early stage esophageal squamous cell carcinoma (ESCC).

**Methods:**

A consecutive series of ninety biopsy samples of esophageal mucosa, collected retrospectively from March 2016 to December 2019, were obtained at Beijing Chao-Yang Hospital, a tertiary care facility in Beijing, China. These samples were categorized into four groups: non-neoplastic squamous lesions (Non-NSL), low-grade dysplasia (LGD), high-grade dysplasia (HGD), and early stage ESCC. Baseline, molecular analyses (p53 by immunohistochemistry and Ki-67 by immunohistochemistry), and staining analyses (hematoxylin & eosin (HE) and periodic acid-Schiff (PAS) were conducted across the categories. The staining protocols included HE, HE + p53 + Ki-67, HE + p53 + Ki-67 + PAS, and HE + p53/PAS + Ki-67/PAS.

**Results:**

Patients with HGD and ESCC were significantly older and had larger lesions. Elevated p53 and Ki-67 mutation rates were observed in HGD and ESCC, while increased PAS positivity was noted in RE and LGD. The p53, Ki-67, and PAS staining results showed mostly no correlation among the four groups. Abnormal Ki-67 basal layer distribution pattern correlated with histological grades, with higher proportions in HGD and ESCC. HE + p53 + Ki-67 + PAS and HE + p53/PAS + Ki-67/PAS demonstrated complete consistency with the reference standard, with weighted κ values of 1. HE + p53 + Ki-67 + PAS and HE + p53/PAS + Ki-67/PAS protocols exhibited 100% accuracy, sensitivity, and specificity for diagnosing ESCC or ESCC combined with HGD, outperforming the other protocols.

**Conclusions:**

Incorporating specific staining protocols, particularly HE + p53 + Ki-67 + PAS and HE + p53/PAS + Ki-67/PAS, enhances the diagnostic accuracy for early stage ESCC, showing promise in advancing the pathology diagnostic pathway.

**Supplementary Information:**

The online version contains supplementary material available at 10.1007/s10388-024-01102-7.

## Introduction

Esophageal cancer, predominantly represented by esophageal squamous cell carcinoma (ESCC), exhibits a dismal prognosis [1, 2]. Due to the asymptomatic nature of early stage ESCC, > 70% of patients are diagnosed with an advanced stage at their first visit and the cancer cells metastasized [3]. Early detection is paramount to improving patient outcomes, with a need for optimizing diagnostic methods. Endoscopy, coupled with Lugol’s iodine staining, is the gold standard for diagnosing precancerous squamous lesions [4], achieving a sensitivity as high as 96% but a specificity of 63% [5]. The acquisition of a precise diagnosis through mucosal biopsy holds paramount significance as it profoundly influences the subsequent treatment strategy, but relying solely on histopathological analysis has limitations. To enhance the positive diagnostic rate, a combination of multiple tumor markers is indispensable [6].

The proliferation cell nuclear antigen Ki-67 is the most widely used marker for cell proliferation activity [7]. p53 is a crucial anti-cancer gene [8]; however, research indicates that the mutation frequency of the p53 gene in malignancies is not excessively high (approximately 50%) [9], and in one study, it has shown that p53 overexpression was present in about 42% of ESCC patients [10, 11]. p53 and Ki-67 expressions are significant indicators of tumorigenesis, associated with diagnosis, prognosis, and treatment response in various cancers, including ESCC [12–15].

The immunohistochemistry (IHC) staining of p53 and Ki-67 is used to diagnose ESCC [16], but the conventional diagnostic pathway is too lengthy, and the low diagnostic efficiency of this pathway, particularly in early stage ESCC, emphasizes the need for refining diagnostic strategies to enhance effective patient management.

Periodic acid-Schiff (PAS) staining is a histological technique used to detect carbohydrates, glycogen, and mucin in tissues [17]. PAS staining can play a role in detecting gastroenterological malignancies [18, 19].

Thus, this retrospective study introduced and assessed the diagnostic potential of advanced staining protocols that include HE, p53, Ki-67, and PAS for identifying early stage ESCC in biopsy specimens.

## Materials and methods

### Study design and patient selection

This retrospective study included a consecutive series of patients diagnosed with ESCC admitted to Beijing Chao-Yang Hospital, Capital Medical University, in Beijing, China, from March 2016 to December 2019.

The inclusion criteria were (1) available comprehensive clinical and pathological data, confirmation of benign mucosal biopsy lesions through clinical follow-up, and confirmation of early ESCC and precancerous lesions through the pathological diagnosis of endoscopic submucosal dissection (ESD) specimens, (2) lesions encompassing early stage ESCC (infiltrating the mucosal lamina propria) and various histopathological lesions, and (3) meticulous efforts to minimize potential influences from sampling and production issues on result interpretation by positioning tissue embedding surfaces upright to ensure the structural integrity of the tissue sections. The exclusion criteria were a history of radiotherapy, chemotherapy, or prior esophageal surgery before undergoing endoscopy. The samples were anonymized and randomly selected from the hospital’s pathology archives to maintain patient confidentiality and mitigate selection bias.

**Fig. 1:**
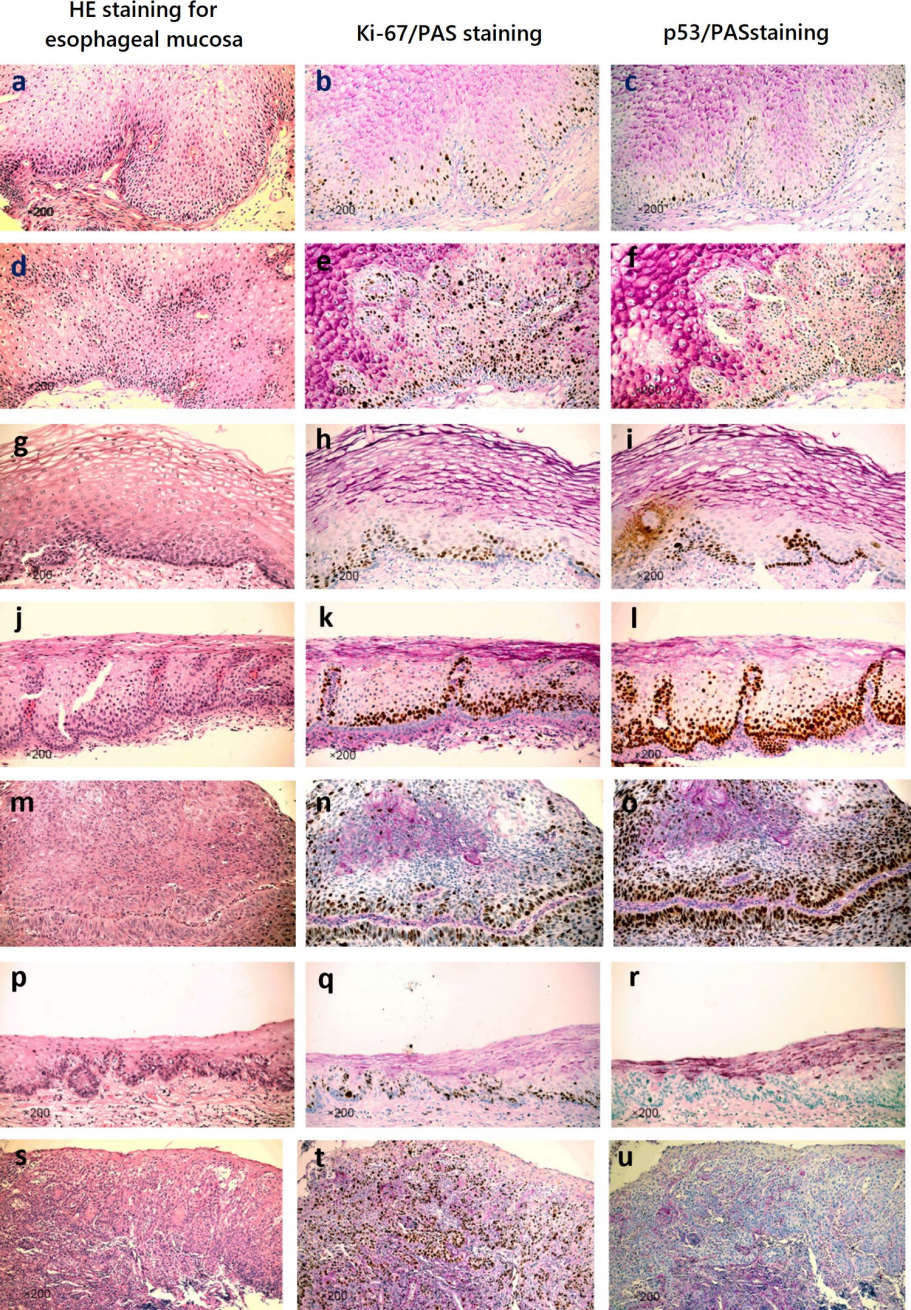
Pathological images obtained from esophageal mucosal biopsy samples, HE staining indicated non-neoplastic squamous lesions (Non-NSL) of esophageal mucosa, low-grade dysplasia (LGD) of esophageal mucosal tissue, high-grade dysplasia (HGD) of esophageal mucosal tissue, and esophageal squamous cell carcinoma (ESCC) of esophageal mucosal tissue. Non-NSL from esophageal mucosa: **a** HE staining illustrating reactive hyperplasia characterized by increased cellular growth. **b** Reactive hyperplastic lesions displaying scattered abnormal Ki-67 expression in basal layer cells, indicating proliferative activity. **c** Detection of p53 protein highlighting its presence in reactive hyperplasia. **d** HE staining showing proliferating papillary structures within the tissue. **e** Proliferating papillary structures displaying scattered abnormal Ki-67 expression in cells around the papillary formations, signifying active cell division. **f** Detection of p53 protein in proliferating papillary structures suggesting cellular abnormalities. LGD of esophageal mucosal tissue: **g**, **j** HE staining indicating dysplastic cells confined within the lower half of the epithelium. **h**, **k** Increased number of abnormal Ki-67 expression cells with a continuous distribution pattern, indicating heightened cellular proliferation. **i**, **l** Remarkable increase in the number of p53-positive cells, suggesting cellular abnormalities associated with dysplasia. HGD of esophageal mucosal tissue: **m**, **p** HE staining showing histological features characteristic of high-grade dysplasia. **n**, **q** Detection of Ki-67 protein indicating increased cellular proliferation. **o**, **r** p53 mutation, signifying abnormal cellular behavior associated with high-grade dysplasia. ESCC of esophageal mucosal tissue: **s** HE staining displaying atypical cells occupying the entire epithelial thickness, indicative of ESCC. **t** Marked increase in the number of abnormal Ki-67 expression cells, highlighting intense cellular proliferation. **u** Complete absence of p53 protein, suggesting abnormal cellular behavior associated with ESCC

The specimens were meticulously classified based on the World Health Organization (WHO) Histological Classification Standard for Digestive System Tumors [20] (Version 5, 2019) into categories, such as non-neoplastic squamous lesions (Non-NSL) (Fig. 1a–f), low-grade dysplasia (LGD) (Fig. 1g–l), high-grade dysplasia (HGD) (Fig. 1m–r), and esophageal squamous cell carcinoma (ESCC) of esophageal mucosal tissue (Fig. 1s–u). According to the Japan Esophageal Society (JES) classification of Esophageal Cancer, the LGD, HGD, and ESCC in the WHO classification were squamous intraepithelial neoplasia (SIN) and squamous cell carcinoma in situ (SCC in situ), and invasive squamous cell carcinoma, respectively [21].

### Data collection and definition

***Tissue preparation and hematoxylin and eosin (HE) staining.*** The specimens were fixed in 10% neutral formalin and underwent dehydration, hyalinization, and wax immersion. The tissues were arranged vertically and embedded, with each slide containing four consecutive 4-μm-thick tissue sections. The HE staining dye solution used in the process was from Beijing Baron Company (Beijing, China), and the staining procedure was performed using a Roche automatic staining and sealing machine (Los Angeles, CA, USA).

***IHC of p53 and Ki-67.*** The expression levels of the p53 and Ki-67 proteins were assessed in early cancerous, precancerous tissues, and non-neoplastic samples. Tissue sections, 4-μm-thick, were stained using a fully automated IHC staining machine (BenchMark ULTRA; Roche, Los Angeles, CA, USA). Reagents, including anti-p53 antibody (DO-7, Roche, USA, lot number: J12042) and anti-Ki-67 antibody (30-9, Roche, USA, lot number: H36867), were used in the experiment. One positive control section was included in each staining batch for each reagent to ensure quality control. Two sections of each protein assay were utilized for every sample and divided into two groups.

***PAS staining.*** The 4-μm-thick tissue sections underwent a series of steps, including baking and dewaxing, followed by PAS histochemical staining using a kit from Zhuhai Beso Co., Ltd., Zhuhai, China, according to the manufacturer’s instructions. Subsequently, the p53 and Ki-67 immunohistochemical sections, which had been previously stained, underwent additional staining using the PAS technique.

***Interpretation standard and methodology.*** The interpretation of HE-stained sections adhered to the esophageal tumor classification and diagnostic criteria outlined in the 5th Edition of the WHO 2019 guidelines [22]. HGD, encompassing carcinoma in situ, was identified based on the fulfillment of any of the following criteria: (1) a high degree of cytologic heterogeneity, irrespective of the extent of epithelial involvement; (2) mild cytological atypia with over 1/2 epithelial involvement; (3) in situ squamous cell carcinoma diagnosed according to the Japanese criteria [21, 23]. LGD was characterized by increased cell density in the lower half of the squamous epithelium with only mild cytological heterogeneity. In a previous expert consensus [24], experts have concluded the characteristics of non-neoplastic lesions (including reflux esophagitis and squamous epithelial dysplasia), and in this study, we performed the discrimination of non-neoplastic lesions from esophageal cancers based on the criteria.

The evaluation of immunohistochemical results focused exclusively on p53 and Ki-67 nuclear staining. Specifically selected control HE-stained sections were used for the percentage count of tumor cell areas. First, tumor cells on the surface layer were excluded and only cells in the middle and lower layers were included for analysis, the evaluation criteria for p53 are as follows: (1) Wild type: ≤ 50% tumor cells show scattered strong positive and weak positive staining; (2) Mutant type: > 50% of tumor cells show strong positive staining throughout the nucleus (missense mutation) or complete loss of expression (nonsense mutation) [25–29]. The Ki-67 assessment criteria were summarized as follows: (1) normal esophageal squamous epithelial basal cells typically show either Ki-67 negativity or only rare scattered positive cells (< 5/HPF, × 400, field diameter 550 μm), (2) parabasal layer cells exhibit active proliferation with a significant expression in bands (> 60%), and (3) the epithelial surface layer remains Ki-67-negative. Abnormal Ki-67 distribution patterns (abnormal phenotypic structures) in the squamous epithelium were defined by the presence of one among (1) a higher number of positive cells in the basal layer (≥ 5/HPF), (2) no or significantly reduced expression of cells in the parabasal layer (< 50%), or an equal or higher number of positive cells in the basal layer compared to the parabasal layer, and (3) the presence of Ki-67 expression in the epithelial superficial layer (> 2 cells/HPF). All IHC sections were appropriately stained, and internal controls were included to ensure accurate evaluation [28]

The PAS staining was also evaluated, and PAS staining exhibited a rose-red coloration of glycogen, facilitating the observation of glycogen distribution in the esophageal mucosal epithelium, since there were no widely recognized criteria for PAS quantification, to further analyze the relation between glycogen change and differentiation degree of esophageal squamous epithelial cells, we took WHO Histological Classification Standard for Digestive System Tumors as a reference, and performed a semi-quantitative evaluation method for PAS staining intensity description, if PAS staining present over 1/2 of the tissue, it is classified to PAS > 1/2; if the PAS staining present in less than 1/2 of the section, it is classified to PAS < 1/2.

The samples were grouped based on the staining method: (1) HE staining; (2) HE staining combined with p53 and Ki-67; (3) HE staining combined with p53, Ki-67, and PAS staining; (4) HE staining combined with p53 and Ki-67 plus PAS staining. Two senior pathologists independently reviewed the slides and engaged in discussions to resolve any disagreements. The clinical follow-up results and the final pathological results of ESD specimens served as the reference standard.

### Statistical analysis

Statistical analyses were conducted using SPSS 23.0 (IBM, Armonk, NY, USA). The continuous variable age followed a normal distribution and was summarized using mean ± standard deviation and compared using a one-way analysis of variance (ANOVA) test. Categorical variables were described as *n* (%), and the Chi-squared test or Fisher’s exact test was used as appropriate.

The diagnostic efficacy was evaluated using sensitivity, specificity, positive predictive value (PPV), negative predictive value (NPV), and accuracy, all derived by comparing the staining results against the reference standard diagnosis. The consistency was assessed using weighted κ statistics. A two-tailed *p* value of < 0.05 was considered statistically significant for all analyses.

## Results

### Demographic and lesion characteristics across different groups

Ninety consecutive biopsy samples of esophageal mucosa were collected, comprising four groups: Non-NSL (*n* = 28), LGD (*n* = 18), HGD (*n* = 32), and early stage ESCC (*n* = 12). Table 1 presents the patient and lesion characteristics. The patients diagnosed with LGD, HGD, and early stage ESCC were significantly older than those with Non-NSL. The lesion size observed in HGD and ESCC was notably larger compared with Non-NSL and LGD. The results showed elevated p53 and Ki-67 positivity in HGD and ESCC. Specifically, all p53 were wild type in the Non-NSL group. Conversely, increased positivity for PAS staining was more frequently observed in Non-NSL and LGD, with PAS-stained glycogen predominantly present above half of the epithelial height in 85.7% of RE and 77.8% of LGD (Table 1).

**Table 1:** Demographic and lesion characteristics across four groups

Variables	Total (*n* = 90)	Histological grades				*p* value^1^
		Non-NSL (*n* = 28)	LGD (*n* = 18)	HGD (*n* = 32)	ESCC (surface) (*n* = 12)	
Sex						0.71
Males	61 (67.8%)	18 (64.3%)	12 (66.7%)	21 (65.6%)	10 (83.3%)	
Females	29 (32.2%)	10 (35.7%))	6 (33.3%)	11 (34.4%)	2 (16.7%)	
Age, years						0.03
Mean (SD)	61.7 (8.7)	57.7 (8.8)	62.9 (9.3)	63.1 (8.0)	65.2 (7.3)	
Esophageal position						0.91
Upper	7 (7.8%)	2 (7.1%)	2 (11.1%)	3 (9.4%)	0 (0.0%)	
Middle	57 (63.3%)	16 (57.1%)	12 (66.7%)	21 (65.6%)	8 (66.7%)	
Lower	26 (28.9%)	10 (35.7%)	4 (22.2%)	8 (25.0%)	4 (33.3%)	
Size of lesion, cm						< 0.001
< 2	45 (50%)	24 (85.7%)	12 (66.7%)	8 (25.0%)	1 (8.3%)	
2–4	21 (23.3%)	4 (14.3%)	5 (27.8%)	9 (28.1%)	3 (25.0%)	
> 4	24 (26.7%)	0 (0.0%)	1 (5.6%)	15 (46.9)	9 (66.7%)	
p53						< 0.001
Wild-type pattern	58 (64.4%)	28 (100%)	10 (56%)	15 (47%)	5 (42%)	
Mutation pattern	32 (35.6%)	0 (0%)	8 (44%)	17 (53%)	7 (58%)	
Ki-67						0.01
Normal pattern	43 (47.8%)	20 (71%)	9 (50%)	11 (34%)	3 (25%)	
Abnormal pattern	47 (52.2%)	8 (29%)	9 (50%)	21 (66%)	9 (75%)	
PAS						< 0.001
< 1/2	46 (51.1%)	4 (14%)	4 (22%)	28 (87%)	10 (83%)	
> 1/2	44 (48.9%)	24 (86%)	14 (78%)	4 (13%)	2 (17%)	

*Non-NSL* Non-neoplastic squamous lesions, *LGD* Low-grade dysplasia, *HGD* High-grade dysplasia, *ESCC* (surface) Esophageal squamous cell carcinoma infiltrating the lamina propria of the mucosa

^1^Fisher’s exact test was performed for categorical variables, and ANOVA was performed for continuous variables

### Correlation of Ki-67 basal layer distribution with histological grades

In Ki-67-positive cases, the distribution of abnormal Ki-67 in the basal layer strongly correlated with the histological grades. An abnormal Ki-67 basal layer indicates the presence of more than or equal to five positive cells per high-power field (≥ 5 cells/HPF) or several basal layer positive cells equal to or greater than those in the parabasal layer. An 81.0–89.9% proportion of abnormal basal layer distribution was observed in HGD and ESCC compared with Non-NSL and LGD (Table 2).

**Table 2:** The correlation between the distribution of Ki-67 positive staining at the basal layer and the histological grades of esophageal mucosal specimens

		Non-NSL	LGD	HGD	ESCC (surface)	*p* value
Abnormal Ki-67	Non-basal layer	5 (62.5%)	7 (77.8%)	4 (19.0%)	1 (11.1%)	0.002
	Basal layer	3 (37.5%)	2 (22.2%)	17 (81.0%)	8 (89.9%)	

*Non-NSL* Non-neoplastic squamous lesions, *LGD* Low-grade dysplasia, *HGD* High-grade dysplasia, *ESCC* (surface) Esophageal squamous cell carcinoma infiltrating the lamina propria of the mucosa

*p* value was estimated using Fisher’s exact test

### Pairwise correlation of p53, Ki-67, and PAS staining among groups

**Fig. 2:**
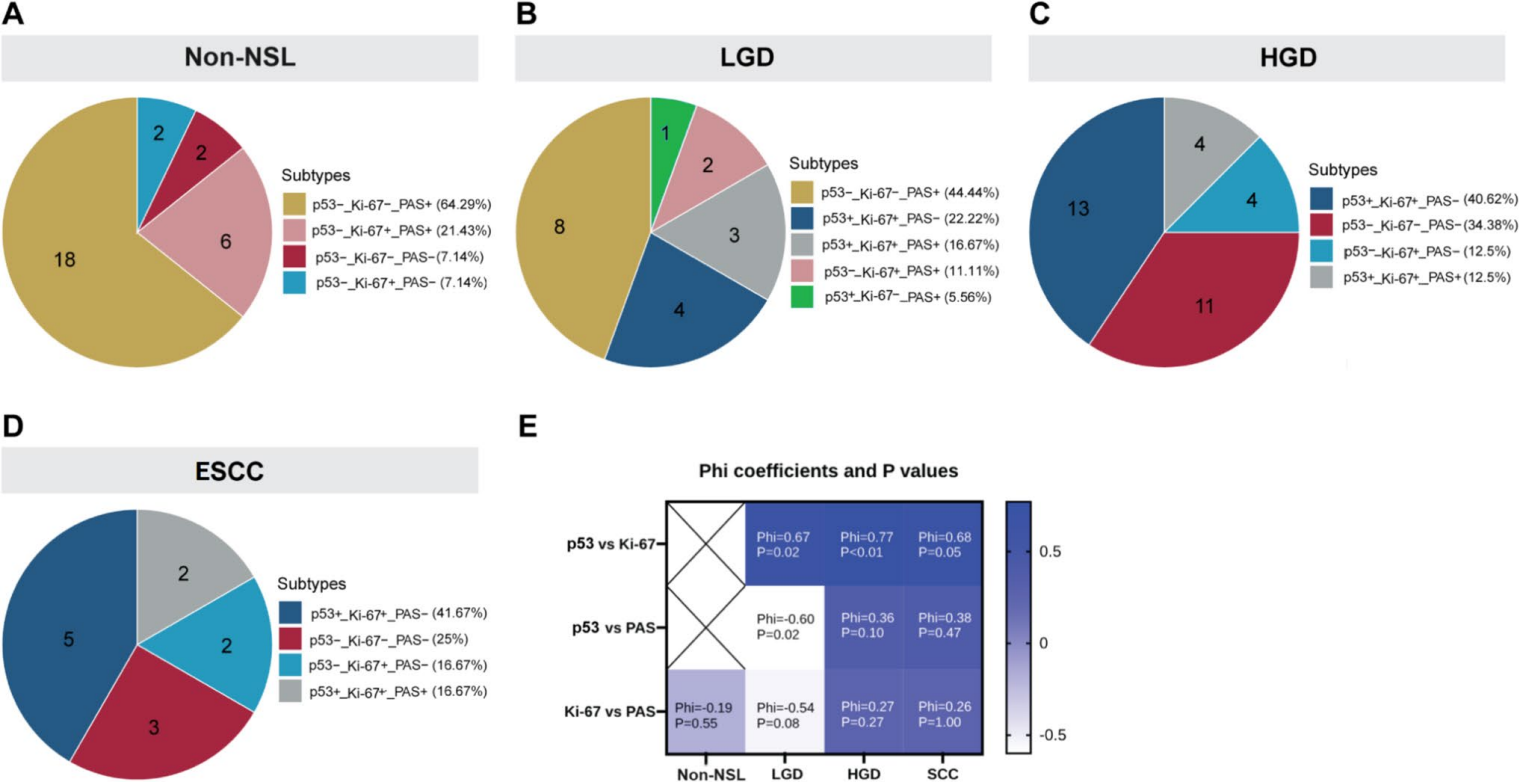
Pairwise correlation and molecular typing distribution of p53, Ki-67, and PAS staining among various groups. **A** Pie charts stained with p53, Ki-67, and PAS in Non-NSL group. **B** Pie plots stained with p53, Ki-67, and PAS in LGD group. **C** Pie charts stained with p53, Ki-67, and PAS in HGD group. **D** Pie plots stained with p53, Ki-67, and PAS in early ESCC group. **E** Correlated heat maps stained with p53, Ki-67, and PAS between the four groups, with color bars representing the size of Phi coefficients. **Note**: p53-/ + represents the wild/mutant type of p53, Ki-67-/ + indicates the normal/abnormal expression of Ki-67, and PAS-/ + indicates that PAS + occupies the epithelial height < 1/2 and > 1/2

The staining results for p53, Ki-67, and PAS displayed minimal correlation among the four groups, suggesting distinct molecular profiles across different stages of esophageal mucosal alteration (Fig. 2). In addition, Spearman correlation analysis showed that p53, Ki-67, and PAS staining showed no correlation between glycogen in Non-NSL, and abnormal expression of p53 and Ki-67 was significantly positively correlated in the process of dysplasia to early carcinogenesis (rs = 0.671, 0.770, 0.683, *p* < 0.05), and p53, Ki-67, and PAS staining were significantly negatively correlated with each other at LGD (rs = −0.598/−0.535, *p* < 0.05), but had no correlation with HGD to early cancer (rs = 0.355, 0.378/0.274, 0.258, *p* > 0.05) (Supplementary Table 1 and 2).

### Comparative performance of staining protocols

When assessing the effectiveness of various staining protocols, the concordance between the diagnosis based on HE staining and the reference standard was the lowest (weighted κ of 0.64), followed by HE + p53 + Ki-67 staining (weighted κ of 0.78). The HE + p53 + Ki-67 + PAS and HE + p53/PAS + Ki-67/PAS protocols exhibited complete consistency with the reference standard, achieving a weighted κ of 1 (Table 3).

**Table 3:** Concordance between the diagnoses obtained from four staining methods and the reference standard

Staining method	Weighted Kappa	95% CI	*p* value
HE	0.64	0.54–0.73	< 0.001
HE + p53 + Ki-67	0.78	0.71–0.85	< 0.001
HE + p53 + Ki-67 + PAS	1.00	–	–
HE + p53 + Ki-67/PAS	1.00	–	–

In diagnosing ESCC or ESCC combined with HGD, the HE + p53 + Ki-67 + PAS and HE + p53/PAS + Ki-67/PAS protocols exhibited 100% accuracy, sensitivity, specificity, PPV, and NPV. These results outperform the other staining protocols used in this study (Tables 4 and 5).

**Table 4:** Performance of four staining methods in the diagnosis of early stage ESCC

Staining method	Sensitivity	Specificity	PPV	NPV	Accuracy
HE	0.83 (0.5,0.98)	1.00 (0.95,1.00)	1.00 (0.69,1.00)	0.97 (0.91,1.00)	0.98 (0.92,1.00)
HE + p53 + Ki-67	1.00 (0.74,1.00)	1.00 (0.95,1.00)	1.00 (0.74,1.00)	1.00 (0.95,1.00)	1.00 (0.96,1.00)
HE + p53 + Ki-67 + PAS	1.00 (0.74,1.00)	1.00 (0.95,1.00)	1.00 (0.74,1.00)	1.00 (0.95,1.00)	1.00 (0.96,1.00)
HE + p53 + Ki-67/PAS	1.00 (0.74,1.00)	1.00 (0.95,1.00)	1.00 (0.74,1.00)	1.00 (0.95,1.00)	1.00 (0.96,1.00)

*PPV* Positive predictive value; *NPV* Negative predictive value

**Table 5:** Performance of four staining methods in the diagnosis of early stage ESCC combined with HGD

Staining method	Sensitivity	Specificity	PPV	NPV	Accuracy
HE	0.82 (0.67,0.92)	0.96 (0.85,0.99)	0.95 (0.82,0.99)	0.85 (0.72,0.93)	0.89 (0.81,0.95)
HE + p53 + Ki-67	0.93 (0.81,0.99)	1.00 (0.92,1.00)	1.00 (0.91,1.00)	0.94 (0.83,0.99)	0.97 (0.91,0.99)
HE + p53 + Ki-67 + PAS	1.00 (0.92,1.00)	1.00 (0.92,1.00)	1.00 (0.92,1.00)	1.00 (0.92,1.00)	1.00 (0.96,1.00)
HE + p53 + Ki-67/PAS	1.00 (0.92,1.00)	1.00 (0.92,1.00)	1.00 (0.92,1.00)	1.00 (0.92,1.00)	1.00 (0.96,1.00)

*PPV* Positive predictive value; *NPV* Negative predictive value

**Fig. 3:**
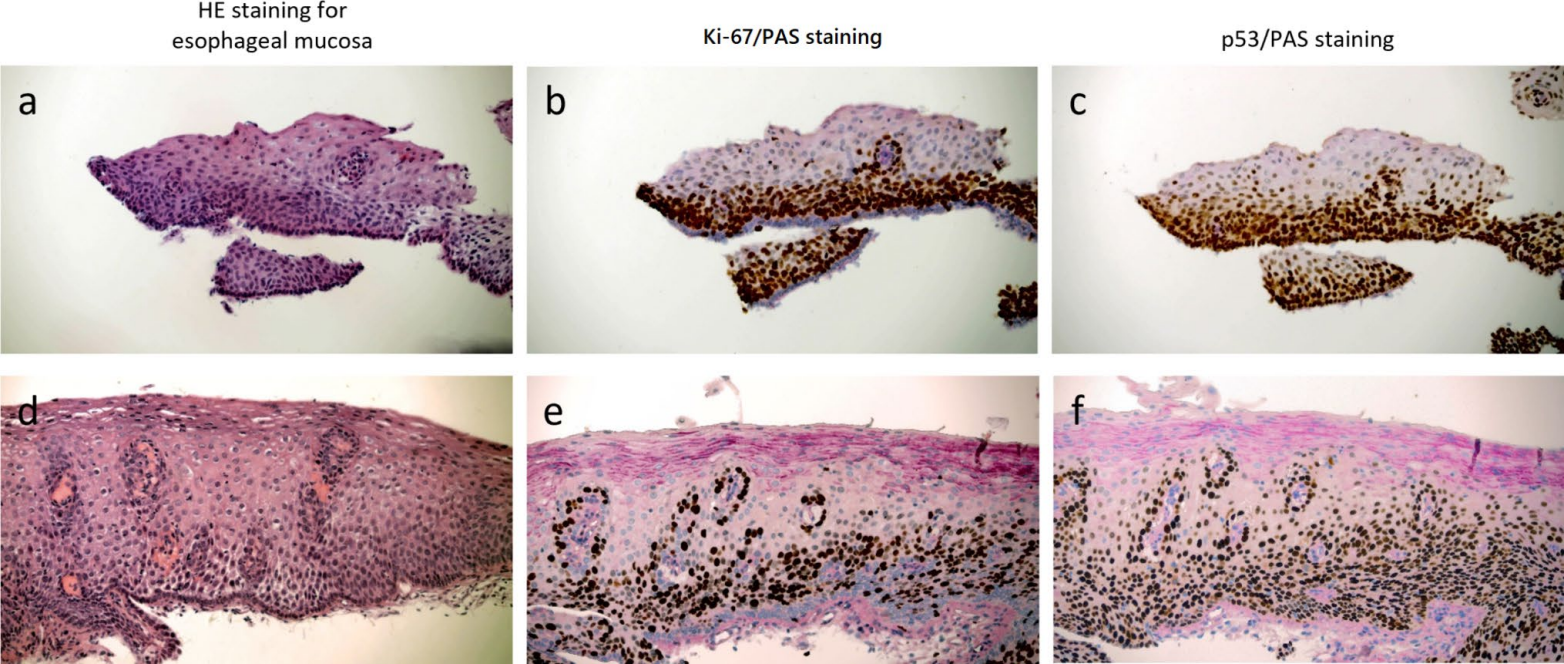
HE staining of epithelial tissue sections from two cases with intact and incomplete low-grade dysplasia (LGD) of esophageal mucosa. The upper part of the epithelial tissue of the esophageal mucosa was missing, and the atypical changes were initially diagnosed by HE staining of the remaining basal cells **a** HE staining showed slight atypia. **b** Ki-67/PAS staining showed abnormal expression of Ki-67 and expression of Ki-67- in basal cells in areas of cytopathic lesions where PAS staining was absent. **c** p53/PAS staining, PAS staining deletion of cytopathic areas, p53 is mutant expression. HE staining of a complete section of esophageal mucosal epithelial tissue was initially diagnosed as atypical cell changes: **d** HE staining showed slight atypia. **e** Ki-67/PAS staining showed abnormal expression of Ki-67 and expression of Ki-67 in basal cells in areas of cytopathic lesions where PAS staining was absent. **f** p53/PAS staining, PAS staining deletion of cytopathic areas, p53 is mutant expression

For details, p53 is not expressed in benign lesions, but during the transition from esophageal squamous epithelial dysplasia to early cancer, p53 expression increases rapidly. Ki-67 has low abnormal expression in benign esophageal squamous epithelial lesions. Ki-67 is closely related to cell synthesis and metabolism and is highly expressed in malignant tumors. This study determined the abnormal expression pattern of Ki-67 in benign lesions. In LGD, the basal layer is dominated by Ki-67- (78%), while in HGD, the basal layer is dominated by Ki-67 + (81%). Furthermore, in early stage cancers, the Ki-67-positive pattern was more common in the basement membrane (89%). PAS staining of esophageal squamous epithelium revealed a pattern of decreased glycogen content in both benign and cancerous lesions. In LGD, positive expression levels of p53 and Ki-67 were limited to 50% or less, and half of the samples could not be evaluated. PAS staining can be diagnosed based on the glycogen content (when the PAS + height exceeds 1/2, the glycogen content is close to 80%) and the scope of the lesion. In high-grade esophageal intraepithelial neoplasia and early esophageal squamous cell carcinoma, the p53 mutation rate is 53–58%, the abnormal distribution pattern of Ki-67 is 66–75%, and the basal cells are mainly Ki-67 + . When HE section was initially interpreted as the more vague result of atypical cell changes, the analysis of p53, Ki-67, and PAS staining could be more accurately interpreted as the basis for dysplasia (Fig. 3).

## Discussion

In this study, we first revealed a correlation between patient age, lesion size, and the severity of esophageal mucosal changes, underlining the importance of considering these factors in the diagnostic process. Within the scope of this study, p53 expression was nonexistent in Non-NSL, while it escalated rapidly during the transition from esophageal squamous epithelial dysplasia to early stage carcinoma. Furthermore, a significant positive correlation between p53 expression and histological grading was observed. These outcomes align with the literature [30], implying a potential correlation between p53 and tumor staging [31]. The presence of point mutations in p53 in LGD was substantiated by Kobayashi et al., elucidating that such mutations embody a neoplastic alteration sharing characteristics with cancerous lesions. While histopathological assessment of HGD remains relatively straightforward, differentiating LGD from reactive atypical epithelia (RAE) poses challenges that p53 mutations can help overcome [32].

The present study revealed a low positive expression of Ki-67 in benign esophageal squamous epithelial lesions, which is consistent with prior research [33]. Furthermore, the aberrant Ki-67 expression exhibits a significant and positive correlation with histological grade, aligning with previous research emphasizing Ki-67 as a pivotal marker of tumor cell proliferation. The association of Ki-67 with cellular synthesis and metabolism and its frequent overexpression in malignant tumors underscores its crucial role in the initiation and progression of esophageal cancer.

Seery et al. categorized cells adjacent to the basement membrane into the basal layer and the parabasal layer. Subsequently, they divided the basal layer into the interpapillary basal layer (IBL) and the papillary basal layer (PBL). In normal esophageal mucosa, the PBL and parabasal cell layer exhibited abnormal Ki-67 expression, while the IBL showed normal Ki-67 expression [34]. This study identified abnormal Ki-67 expression patterns in benign lesions. However, in LGD, the basal layer predominantly demonstrated the normal Ki-67 pattern (78%), while in HGD, the basal layer predominantly displayed an abnormal Ki-67 pattern (81%). Furthermore, in early stage cancer, the abnormal Ki-67 pattern was more prevalent in the basal lamina (89%). These results contradict a previous study that differentiated between normal/RAE and LGD based on the strength of the abnormal expression level of Ki-67 but failed to distinguish between LGD and HGD [35]. This discrepancy may be attributed to variations in methods used to analyze abnormal expression patterns of Ki-67 across different studies. The method applied in this study is more objective, although it has been less frequently reported in the literature.

In this study, PAS staining of the esophageal squamous epithelium unveiled a diminishing pattern in glycogen content across benign and cancerous lesions. These findings are consistent with the outcomes of a prior study conducted by Bolidong et al. [36], where a pronounced loss of cellular glycogen in low-grade dysplasia persisted throughout the invasive ESCC stages, indicating potential impairments in glycogen synthesis and/or excessive breakdown during ESCC development and progression.

The correlation analysis involving p53, Ki-67, and PAS special staining revealed no significant correlations in Non-NSL. Still, a notable positive correlation surfaced between p53 and Ki-67 expression in squamous epithelial dysplasia and early stage carcinoma. This finding demonstrates greater robustness compared to the study by Sasano et al. [37], which indicated a positive correlation between p53 and Ki-67 expression in precancerous esophageal lesions. Furthermore, the present investigation unveiled p53 and Ki-67 expression showed distinctly negative correlation with PAS staining intensity in LGD patients, with no correlation observed in the other stages. Consequently, in diagnosing early stage ESCC, the principal role of PAS special staining lies in determining the localization and extent of lesion cells, with the indication of glycogen deficiency being of secondary importance.

This study introduced an innovative diagnostic methodology by integrating PAS staining with p53 and Ki-67 alongside HE staining, a combination not hitherto reported. The findings revealed that the HE + p53 + Ki-67 + PAS and HE + p53/PAS + Ki-67/PAS staining protocols exhibited a high concordance with the ultimate pathological diagnosis in esophageal biopsy specimens.

Despite incorporating p53 and Ki-67 biomarkers into the HE + p53 + Ki-67 staining method, discrepancies arose. First, determining lesion extent solely through cellular atypia and structural changes in HE staining is challenging [38]. While p53 and Ki-67 markers could assist in the analysis, their specificity was low and lacked correlation. Incorporating PAS staining, revealing the papillary structure, and offering insights into the extent of lesion cells proved beneficial in mitigating diagnostic challenges. Second, diagnosing cases of LGD in HE staining is subjective, making them prone to misdiagnosis. Kobayashi et al. [32] highlighted significant differences between LGD and RAE, emphasizing nuclear size and shape over nuclear density and occupancy.

The presence of mutant p53 serves as a distinguishing factor for distinguishing RAE, whereas the anomalous basal cell distribution pattern of Ki-67 is indicative of HGD, aligning with the observations made by Hou et al. This subtype is characterized by mild cytological heterogeneity and extension into the lower region of the squamous epithelium. In such cases, the differential diagnosis hinges upon assessing the expression of p53 mutants and recognizing the abnormal distribution pattern of Ki-67 [28].

Nevertheless, this study showed that positive expression levels of p53 and Ki-67 in LGD were limited to 50% or below, with half of the samples eluding evaluation. The use of PAS staining, offering valuable insights for diagnosing samples based on glycogen percentage (close to 80% when exceeding 1/2) and lesion extent delineation, enhances the feasibility of diagnosing such samples. Notably, several instances of LGD manifested a glycogen loss rate surpassing 1/2, notwithstanding the presence of only mild cellular atypia. Pathological analysis of the corresponding ESD specimens substantiated high-grade lesions or carcinomas, signifying an active proliferation of lesion cells with a progressive inclination.

In instances of HGD and early stage cancerous lesions, both cellular and structural atypia were notably discernible. Nevertheless, the heterogeneous nature of tumor cell atypia posed challenges, especially in cases displaying a high degree of differentiation, wherein tumor cells closely resembled mid-surface cells of the multilayer squamous epithelium. This resemblance added complexity to pathological diagnoses [39]. Despite severe nucleus atypia, these cases were susceptible to misdiagnosis as LGD or RAE in biopsy specimens due to discontinuous histology [40]. In instances of HGD and early stage ESCC, frequent observations revealed p53 mutation rates ranging from 53 to 58% and abnormal distribution patterns of Ki-67, notably appearing as abnormal distribution patterns of Ki-67in basal cells, ranging from 66 to 75%. The combination of p53, Ki-67, and PAS staining emerged as a valuable adjunct to the aforementioned indices, particularly when combined with PAS special staining, which emphasized the extent of the lesion. Both HE + p53 + Ki-67 + PAS and HE + p53/PAS + Ki-67/PAS staining methodologies efficiently diagnose early stage ESCC lesions in esophageal biopsy specimens, with the latter requiring fewer slides (4/3). It indicates a potential reduction in the workload for pathologists through this combined staining approach. The high accuracy and reliability of these combined protocols also suggest their potential as the standard diagnostic approach for the early detection of ESCC, particularly in high-prevalence regions such as China.

Nonetheless, it is crucial to acknowledge the study’s limitations. Firstly, the sample size was relatively small, necessitating further research with a larger sample size to validate these findings. Second, the study was conducted retrospectively at a single center, necessitating future prospective studies involving multiple centers for comprehensive validation.

## Conclusion

In conclusion, this study advocates for the incorporation of specific staining protocols, namely HE + p53 + Ki-67 + PAS and HE + p53/PAS + Ki-67/PAS, in the diagnostic pathway for early stage ESCC in esophageal biopsy specimens. The findings of this study underscore the potential of these innovative protocols to enhance diagnostic accuracy significantly, thereby offering a valuable contribution to the field of pathology and improving outcomes for individuals with ESCC. These results warrant further confirmation through prospective, multicenter studies with a larger sample size.

## Supplementary Information

Below is the link to the electronic supplementary material.

Supplementary file1 (DOCX 19 KB)

## Data Availability

All data generated or analyzed during this study are included in this published article.
