# Supplementary material for: p53 and Ki-67 combined with periodic acid-Schiff staining for the diagnosis of early stage esophageal squamous cell carcinoma lesions in biopsy specimens
Source: Esophagus. 2024 Dec 23;22(2):228–38. doi: 10.1007/s10388-024-01102-7 (PMC11929734; doi:10.1007/s10388-024-01102-7)
Supplement: Supplementary file 1 — Supplementary file1 (DOCX 19 KB) [file 10388_2024_1102_MOESM1_ESM.docx]

**Supplementary Table 1** Correlation of p53 and Ki-67 expression in early esophageal squamous cell carcinoma and precancerous lesions

|  | p53 | n | Ki-67 | |
| --- | --- | --- | --- | --- |
|  |  |  | - | + |
| Non-NSL | - | 28 | 20 | 8 |
|  | + | 0 | 0 | 0 |
| LGD | - | 10 | 8 | 2 |
|  | + | 8 | 1 | 7 |
| HGD | - | 15 | 11 | 4 |
|  | + | 17 | 0 | 17 |
| ESCC-surface | - | 5 | 3 | 2 |
|  | + | 7 | 0 | 7 |

**Supplementary Table 2** Correlation of p53, Ki-67 and PAS staining in early esophageal squamous cell carcinoma and precancerous lesions

|  | PAS+ | n | p53 | | Ki-67 | |
| --- | --- | --- | --- | --- | --- | --- |
|  |  |  | - | + | - | + |
| Non-NSL | <1/2 | 4 | 4 | 0 | 2 | 2 |
|  | >1/2 | 24 | 24 | 0 | 18 | 6 |
| LGD | <1/2 | 4 | 0 | 4 | 0 | 4 |
|  | >1/2 | 14 | 10 | 4 | 9 | 5 |
| HGD | <1/2 | 28 | 15 | 13 | 11 | 17 |
|  | >1/2 | 4 | 0 | 4 | 0 | 4 |
| ESCC-surface | <1/2 | 10 | 5 | 5 | 3 | 7 |
|  | >1/2 | 2 | 0 | 2 | 0 | 2 |
